# Increased susceptibility to the face pareidolia illusion in Visual Snow syndrome

**DOI:** 10.1177/03010066251387849

**Published:** 2025-10-27

**Authors:** Blake W. Saurels, Amanda K. Robinson, Jessica Taubert

**Affiliations:** 1 The School of Psychology, The University of Queensland, St Lucia, QLD, Australia

**Keywords:** visual processing, cortical hyperexcitability, illusory faces, face detection

## Abstract

Visual Snow (VS) syndrome is a neurological condition characterised by the constant perception of small flicking dots across the visual field. These symptoms are thought to be caused by hyperexcitability in the visual cortex. This study examined the potential link between VS and susceptibility to the face pareidolia (FP) illusion, where faces are perceived in inanimate objects. Using a self-report VS questionnaire and a standard FP sensitivity task, we collected data remotely from 132 individuals with VS and 104 age-matched controls. Results revealed higher FP sensitivity in individuals with VS, amplified in those with co-occurring migraines. Non-parametric analyses confirmed elevated face scores for VS participants, even when migraines were excluded. A rank-order analysis showed consistency in response patterns across groups, ruling out the idea that extraordinary responses to one stimulus drove the group difference. These findings suggest that individuals with VS syndrome have an increased susceptibility to the FP illusion. Future research should investigate whether hyperexcitability in the visual cortex is the cause.

## Introduction

Visual Snow (VS) syndrome is a neurological condition whereby individuals see static or snow across the entire visual field for a period of no less than three months (Kondziella et al., 2020; Montoya et al., 2024). Surprisingly, the prevalence of VS syndrome in the general population remains unknown, in part because diagnosis is dependent on self-report, making VS impossible to diagnose in pre- or non-verbal individuals. Further, individuals who have experienced the symptoms of VS syndrome from birth or early childhood are unlikely to recognise the symptoms as abnormal. Thus, despite the disturbing and disruptive nature of its defining characteristics (Solly, Clough, Foletta et al., 2021), the prevalence of VS syndrome has proven difficult to estimate and little is understood about the underlying causes (Klein & Schankin, 2021; Silva & Puledda, 2023). A predominant theory posits that VS is caused by hyperexcitability in the visual cortex, affecting how visual input is processed (Aeschlimann et al., 2024; Montoya et al., 2023; Silva & Puledda, 2023; Stern & Robertson, 2024). An eye tracking study has shown that VS syndrome affects attentional shifts and saccade generation (Solly, Clough, McKendrick et al., 2021); individuals with VS syndrome made more erroneous saccades towards peripheral targets. The authors concluded this abnormal viewing behaviour was consistent with heightened activity in the visual cortex.

**Figure 1.**
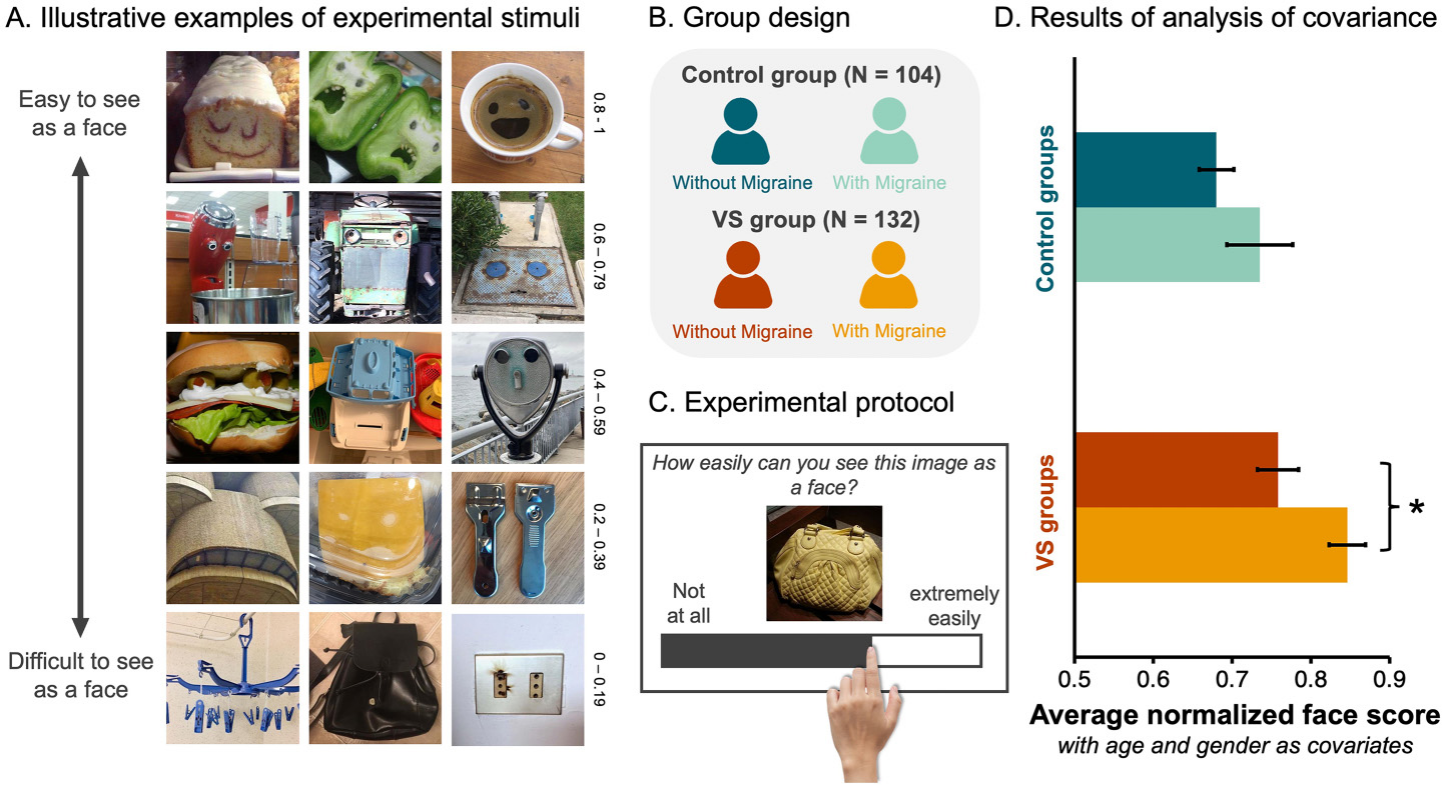
Participants with VS Give Higher Face Scores to Objects. (A) Examples of the 288 non-face objects employed in the FP task. These images vary in how easily they are perceived as faces. For example, the images in the top row represent those given high face scores (i.e., 0.8–1) in a previous study (Wardle et al., 2022), indicating they were very easy to see as faces. Whereas the images in the bottom row represent those given low face scores (i.e., 0–0.19) in a previous study (Wardle et al., 2022), indicating they were difficult to see as faces. (B). Participants that completed more than half of the trials were allocated to one of four groups based on their responses to a VS questionnaire (Kondziella et al., 2020). (C) Illustrative example of an experimental trial. (D) A bar graph visualising the average normalised face scores as a function of participant group (*Dark blue* = control group without migraine, *light blue* = control group with migraine, *dark red* = VS group without migraine, and *orange* = VS group with migraine). Error bars = ±SE. Asterisks flag the significant pairwise differences between the groups with and without migraine (i.e., *p* < .05, after Bonferroni adjustment).

Face pareidolia (FP) is a complex visual illusion which occurs when people see illusory faces in non-face objects (Meng et al., 2012; Omer et al., 2019; Rekow et al., 2021; Rekow et al., 2022; Taubert et al., 2017). Importantly, neural models have suggested the FP illusion stems from an error in the initial processing of coarse visual input by subcortical structures (Hadjikhani & Åsberg Johnels, 2023; Taubert et al., 2018; Taubert et al., 2022a). Consequently, examples of FP spontaneously summon eye movements when presented in the periphery, just like real human faces (Saurels et al., 2024; Stuart et al., 2025; Taubert et al., 2017). Single-unit recordings and E/MEG studies have revealed this initial mistake that is later resolved by slower, more detailed processing of visual features in the visual cortex (Decramer et al., 2021; Koyano et al., 2025; Rekow et al., 2022; Robinson et al., 2025; Sharma et al., 2024; Wardle et al., 2020). In sum, examples of FP are recognised as faces *prior* to being recognised as objects (Caruana & Seymour, 2021; Jakobsen et al., 2023; Keys et al., 2021; Saurels et al., 2024). It follows that when processing in the visual cortex is compromised by hyperexcitability, sensitivity to the FP illusion could be increased, either because the ability to resolve an initial error is stifled or because an initial error is erroneously amplified. Therefore, we predicted that individuals with VS syndrome would be more sensitive to the FP illusion and would rate objects as being more easily seen as faces, than control participants. In this study, we tested this prediction by comparing sensitivity to the FP illusion in two groups of people. To do this, we combined two previously validated measures; (1) a self-report VS questionnaire (Kondziella et al., 2020) and (2) a FP sensitivity task that requires participants to report how easily they can see a face in ambient pictures of non-face objects (Lipp & Taubert, 2024; Robinson et al., 2025; Taubert et al., 2023; Uchiyama et al., 2012; Wang et al. 2023; Wardle et al., 2022) (Figure 1A).

## Methods

### Participants

All procedures were approved by the University of Queensland Human Research Ethics Committee (Protocol number – 2024/HE000324). We recruited a total of 592 volunteers online. However, to ensure the quality of the data, we pre-registered exclusion criteria (AsPredicted #168687). Accordingly, participants were excluded if they: (1) provided incorrect responses to the 32 catch trials, and/or (2) responded to too few trials (i.e., less than 50% of the 320 total trials). Based on these criteria, we removed 301 participants from the sample. We removed another 23 participants because they reported being too young to consent (i.e., younger than 18 years of age), and two others because they were identified as being outside the sample's age range (i.e., older than the average age by more than three *SD*s). Of the 266 participants remaining in the sample, 186 reported having VS but only 132 could be allocated to the VS group according to the diagnostic criteria [1]. In sum, the analysis included 132 participants with VS syndrome, and 104 age-matched controls (Figure 1B). Additionally, since it is not yet clear how VS relates to the disease of migraine (Barrachina-Esteve et al., 2024; Chojdak-Łukasiewicz & Dziadkowiak, 2024; Kondziella et al., 2020; Schankin & Goadsby, 2015; Solly, Clough, Foletta et al., 2021), we allocated all remaining participants to one of four unique groups (VS with migraine, VS without migraine, control with migraine, and control without migraine; Figure 1B) and imposed a 2 × 2 factorial structure on the experimental design with two between-subjects factors (i.e., VS and migraine) each with 2 levels. The age and gender information for each group are available in Table 1 and are employed as covariates in the main analysis. All data collected, including data not analysed here, are available via the open science framework (https://osf.io/hc5b3/).

**Table 1.** Summary of Demographic Information.

				Age range	
Group	Gender	*N*	Mean age (±*SD*)	Minimum	Maximum
Control without migraine	Males	29	35 (±14)	19	67
	Females	51			
	Other	2			
Control with migraine	Males	8	34 (±12)	20	58
	Females	14			
	Other	0			
VS without migraine	Males	24	31 (±10)	18	54
	Females	31			
	Other	3			
VS with migraine	Males	12	33 (±11)	18	62
	Females	59			
	Other	3			

### Procedure

Participants were full volunteers who responded to a recruitment flyer posted in a variety of online support groups and social media pages. The experiment was generated using Qualtrics XM software (https://www.qualtrics.com/en-au/). After completing the VS questionnaire (Kondziella et al., 2020) and some demographic questions (i.e., biological age), every participant was asked to give 320 images a face score, from 0 to 100, using an unmarked sliding bar that was always positioned at zero at the beginning of a trial (Figure 1C). The participants were instructed that if the image was extremely easy to see as a face, they should drag the bar to the right, whereas if they could not see a face in the image, they should drag the bar to the left (Figure 1C). The 320 images were presented one at a time and in a randomised order. Images remained on the screen until the participant responded. Thirty-two of the 320 images contained human faces – these served as catch trials. Participants needed to give these images an average score of 90 or higher to demonstrate they understood the instructions and the task at hand. The inclusion of catch trials was to ensure the quality of the online data, given the remote status of participants and the capacity for distraction (Grootswagers, 2020; Lefever et al., 2007). In this experimental context, a small number of trials containing human faces has served as an appropriate “catch” for inattentive human participants because there is a ground truth; human faces should be easily seen as faces (Taubert et al., 2017; Taubert et al., 2023; Wardle et al., 2020; Wardle et al., 2022). We note that even if the inclusion of 32 human face trials biased responses in a particular direction, there is no reason to believe that they disproportionately affected any of the groups, as all participants responded uniformly to these trials (see Table 2 for results). However, because these responses do not speak to the strength of the FP illusion, the 32 real face trials were removed from all subsequent analyses.

**Table 2.** Average Scores to Catch Trials Across Four Unique Groups.

Group	Mean	SE
Control without migraine	99.7	0.14
Control with migraine	99.9	0.05
VS without migraine	99.7	0.13
VS with migraine	99.7	0.15

### Data Processing

All analyses were performed using SPSS software (version 29.0.1.0), and data visualisations were generated using Matlab software (version R2023_b). For every participant, we first normalised their face scores using the min-max method, and then we computed their average normalised face score across the 288 object trials. Next, we analysed average face scores using a 2 (VS group) × 2 (migraine group) between-subjects analysis of covariance (ANCOVA) with participant age and gender (other = 0, female = 1 and male = 2) entered as covariates.

## Results

When using participants as the unit of analysis, the grand average normalised face score was 0.76 (*SD* = 0.21), indicating that the distribution was skewed. We tested normality using a Kolmogorov–Smirnov test (*D*_236_ = 0.183, *p* < .001) but given that ANCOVA is robust to violations of normality, we proceeded with the statistical analysis. We found main effects of VS (*F*_1,230_ = 10.39, *p* = .001, $\eta_{p}^{2}$ = 0.04) and migraine (*F*_1,230_ = 6.09, *p* = .01, $\eta_{p}^{2}$ = 0.03) group, after adjusting for age (*F*_1,230_ = 4.06, *p* = .05, $\eta_{p}^{2}$ = 0.02) and gender (*F*_1,230_ = 4.94, *p* = .03, $\eta_{p}^{2}$ = 0.02). This indicated that the individuals with VS were more sensitive to the FP illusion than the individuals without VS. Consistent with the results of a previous study (Akdeniz, Gumusyayla et al., 2020), these results also indicate that migraineurs were more sensitive to the FP illusion than the individuals who did not report experiencing migraines.

Although there was no evidence of an interaction between the VS and migraine group factors (*F*_1,230_ = 0.32, *p* = .57, $\eta_{p}^{2}$ = 0.001), we ran two planned contrasts to determine whether there was evidence that the migraine condition had the same impact on participants with and without VS. We found that, within the VS group, average face scores were higher for individuals with migraine (*M* = 0.85, *SE* = 0.02), than for individuals without migraine (*M* = 0.76, *SE* = 0.03; *t*_230_ = 2.54, *p* = .02, adjusted using the Bonferroni rule; Figure 1D). However, we found no evidence that, within the no VS group, such a difference existed (Figure 1D). In sum, while the symptoms of VS and migraine were both associated with higher face scores for objects, we observed that individuals reporting both conditions had the highest susceptibility to the FP illusion.

**Figure 2.**
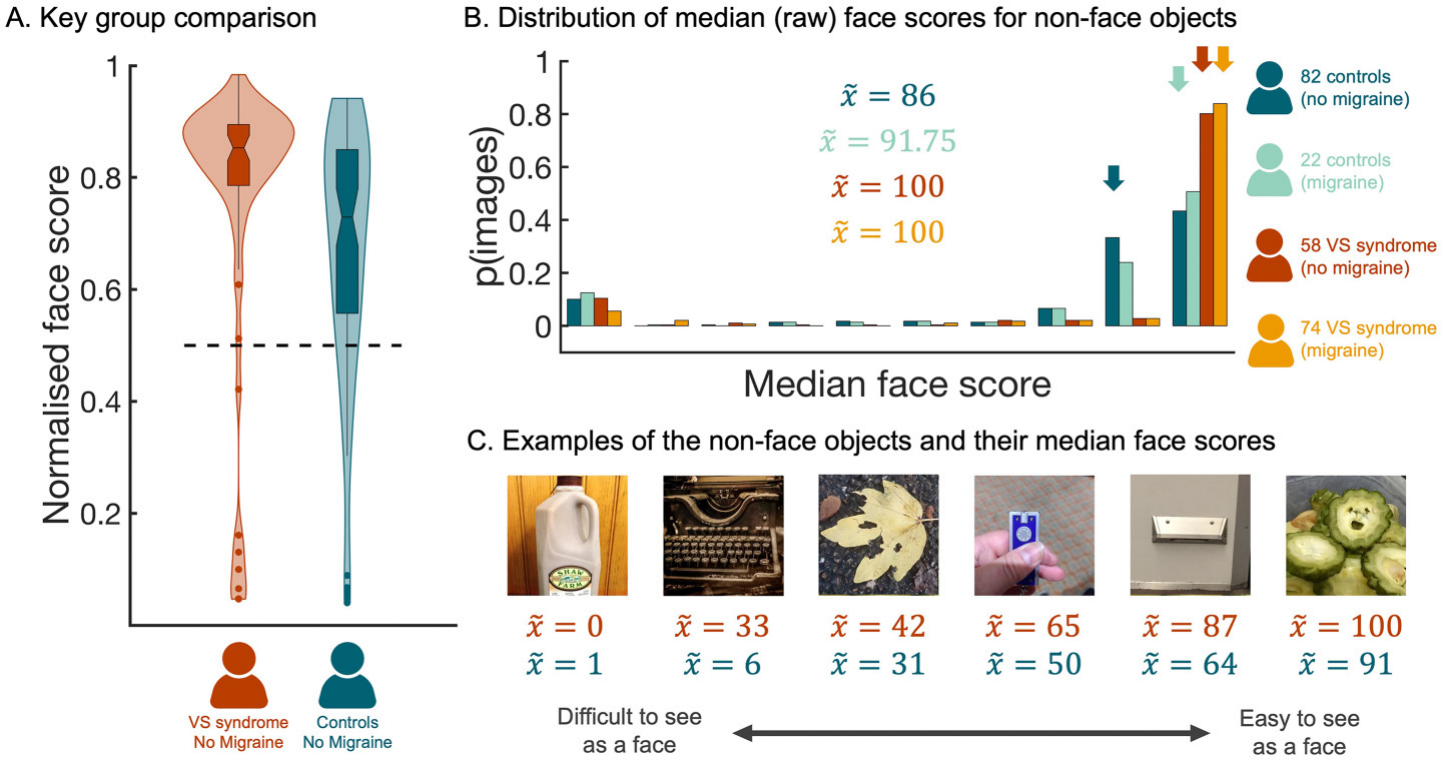
Higher Face Scores When Non-Face Objects are Rated by People with VS. (A) The distribution of normalised faces scores for participants in the VS (without migraine) group and the control (without migraine) group. The horizontal dashed line marks the numerical mid-point on the scale. (B) A histogram showing the distribution of median face scores for the 288 non-face objects. This distribution was plotted separately for each participant group; *dark blue distribution* =control group (without migraine), *light blue distribution* =control group (with migraine), *dark red distribution* = VS group (without migraine), and *orange distribution* = VS group (with migraine). Arrows indicate the median scores for the four groups. (C) Illustrative examples of non-face objects that vary in the median face score allocated to them by participants in the VS without migraine group. Red text provides median score allocated by participants in the VS without migraine group, whereas blue text provides the corresponding median score allocated by participants in the control without migraine group. All data are freely available to the scientific community via the Open Science Framework (https://osf.io/hc5b3/).

Although the main comorbidity of VS is migraine, there is some evidence that these are independent conditions (Akdeniz, Gumusyayla et al., 2020; Barrachina-Esteve et al., 2024; Solly, Clough, Foletta et al., 2021). Thus, as part of our pre-registered analysis plan, we excluded all participants with migraine and confirmed using a non-parametric approach that individuals in the putative VS group gave higher scores to non-face objects (*N* *=* *58, median face score* *=* *0.85*) than individuals in the control group (*N* *=* *82, median face score* *=* *0.73*; Mann–Whitney *U* test, *N* = 140, *Z* = 3.37, *p* < .001, two-tailed; Figure 2A).

To further probe the differences across groups, we ran a complementary analysis using image as the unit of analysis (Taubert et al., 2023). To this end, we computed the median raw face score every image, separately for each of the four participant groups, and compared the corresponding distributions (Figure 2B). This approach uncovered evidence that the distribution of face scores for images of non-face objects differed depending on group (independent samples Kruskal–Wallis test, *N* = 1152, *df* = 3, *H* = 461.34, *p* < .001, two-tailed). Subsequent pairwise contrasts indicated that, although there was no evidence of a difference between the distributions associated with the two VS groups ( $\tilde{x}\;(\mathrm{VS}\;\mathrm{with}\;\mathrm{migraine})$ =100; $\tilde{x}\;(\mathrm{VS}\;\mathrm{without}\;\mathrm{migraine})$ = 100; *U* = 58.23, *p* = .18, adjusted for multiple comparisons using the Bonferroni rule), face scores for non-face objects were higher when rated by individuals in either VS group than when rated by individuals in the control with migraine group ( $\tilde{x}\;(\mathrm{control}\;\mathrm{with}\;\mathrm{migraine})$ = 91.75; control with migraine vs. VS with migraine *U* = 487.8, *p* < .001, Bonferroni adjusted; control with migraine vs. VS without migraine *U* = 429.57, *p* < .001, Bonferroni adjusted; or the control without migraine group ( $\tilde{x}\;(\mathrm{control}\;\mathrm{without}\;\mathrm{migraine})$ = 86, control without migraine vs. VS with migraine *U* = 362.89, *p* < .001, Bonferroni adjusted; control without migraine vs. VS without migraine *U* = 304.66, *p* < .001, Bonferroni adjusted. Finally, there was evidence that face scores for non-face objects were higher when rated by individuals in the control with migraine group than when rated by individuals in the control without migraine group (*U* = 124.91, *p* < .001; Bonferroni adjusted). Examples of the objects with the median face scores given to them by people with VS syndrome are provided in Figure 2C.

**Figure 3.**
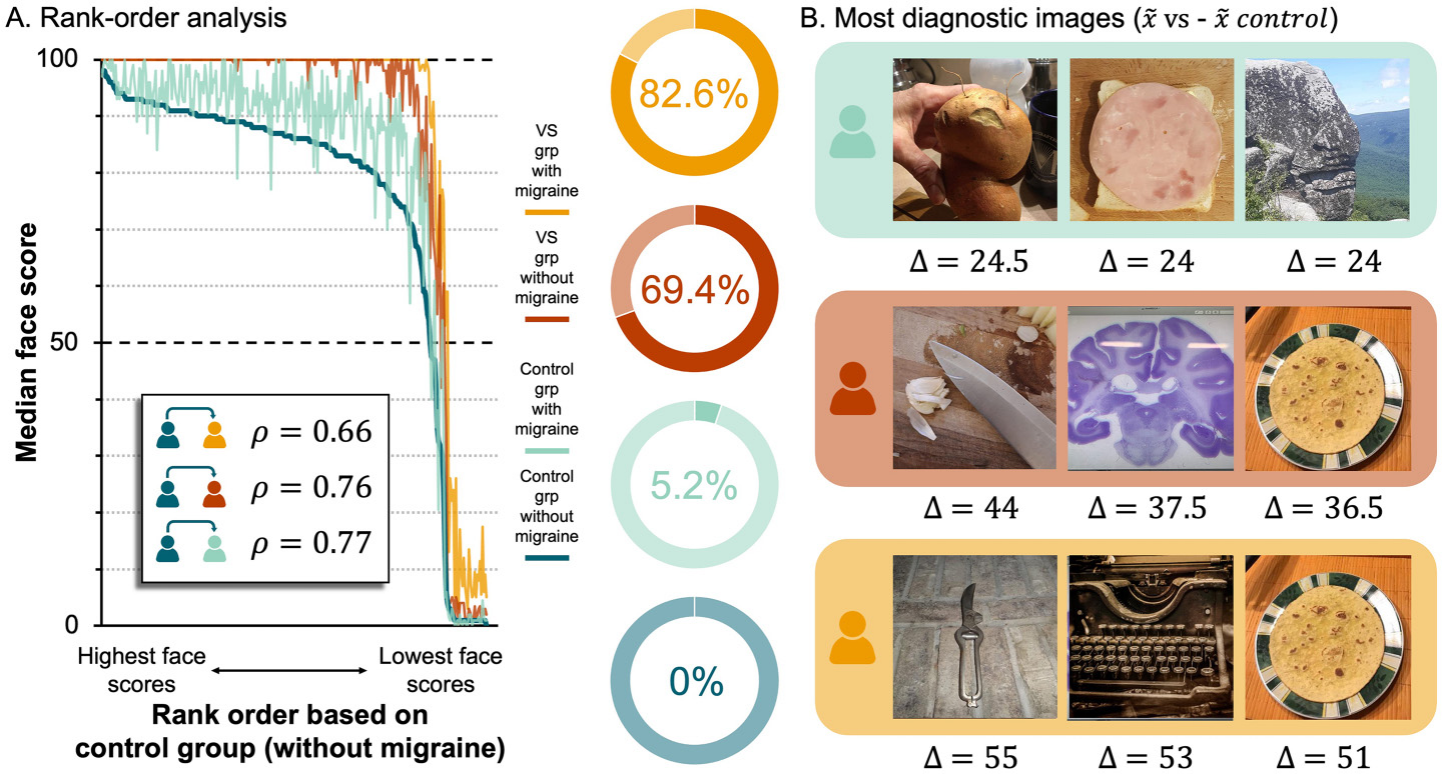
A Rank Order Analysis Yields the Most Diagnostic Non-Face Objects. (A) *Left,* line plot of median face scores across non-face objects, rank ordered based on data from control (no migraine) group. Lines represent groups: *dark blue* = control without migraine, *light blue* = control with migraine, *red* = VS without migraine, *yellow* = VS with migraine. Rho values ( ρ ) are provided in the floating box and indicate the strength of the relationship between groups. *Right,* pie charts showing the proportion of non-face objects with a median face score equal to 100 (i.e., the highest possible face score) as a function of group. Legend shows relationship between group and colour for the line graph (left) and pie charts (right). (B) The three non-face objects associated with the largest differences in face scores across groups (*top row,* $\tilde{x}$ control with migraine – $\tilde{x}$ control without migraine; *middle row,* $\tilde{x}$ VS without migraine – $\tilde{x}$ control without migraine, *bottom row,* $\tilde{x}$ VS with migraine – $\tilde{x}$ control without migraine).

Next, we performed a rank order analysis to determine whether the pattern of responses across non-face objects was consistent across groups. To this end, we reordered the non-face objects from the highest to the lowest median score, based on the data from the control group that reported no VS symptoms or migraines (i.e., control without migraine). Then we computed three Spearman's rank correlation coefficients to estimate the strength of the relationship between this control group and the three other groups (Figure 3A). This revealed significant positive relationships, with the strongest being between the two groups with no VS symptoms (control group without migraine vs. VS group with migraine, ρ = 0.66, *df* = 286, *p* < .001, two-tailed; control group without migraine vs. VS group without migraine, ρ = 0.76, *df* = 286, *p* < .001, two-tailed; control group without migraine vs. control group with migraine, ρ = 0.77, *df* = 286, *p* < .001, two-tailed. Collectively, these findings confirm that, although the face scores were typically higher when sampling people with VS, the pattern of face scores across images was reliably reproduced across groups.

Lastly, to identify the images that evoked the largest differences in scores across groups, we calculated the following differences for every non-face object: (1) $\tilde{x}$ control with migraine – $\tilde{x}$ control without migraine, (2) $\tilde{x}$ VS without migraine – $\tilde{x}$ control without migraine and (3) $\tilde{x}$ VS with migraine – $\tilde{x}$ control without migraine (Figure 3B). As such, the images with the largest differences were the images that best predicted group allocation. To test this idea, for the 58 participants in the VS without migraine and the 82 participants in control without migraine groups, we calculated the average score given to the three images with the largest difference scores (i.e., the images in Figure 3B, middle row). The average score for these three images was above 50 (i.e., the midpoint on the scale) for 67% of the individuals with VS. In contrast, the average score for the same three images was above 50 for only 44% of the controls. Although results require verification using an independent sample, they underscore the potential clinical relevance of the FP illusion as a tool for identifying cases of VS syndrome. Indeed, given the development of techniques to measure sensitivity to FP in both children (Flessert et al., 2022; Wardle et al., 2023) and young infants (Rekow et al., 2021), it may be possible that FP would be leveraged to diagnose VS syndrome in younger, preverbal populations or non-verbal clinical populations.

## Discussion

The results of this study indicate that people with VS are more susceptible to the FP illusion; without any possible prior knowledge of the experimental hypotheses, people with VS syndrome reported greater ease seeing illusory faces in otherwise inanimate objects than people without VS. This effect was amplified in migraineurs, with the people allocated to the group with VS *and* migraine giving significantly higher face scores to non-face objects than the people allocated to the group with VS alone (Figure 1D). This increased sensitivity to FP in migraineurs is consistent with previous research showing that the FP illusion evokes a different pattern of brain activity from migraineurs compared to controls (Akdeniz, Gumusyayla et al., 2020). However, it is not yet clear whether migraine should be considered a symptom of VS or, alternatively, whether migraine and VS syndrome should be considered separate neurological conditions (Barrachina-Esteve et al., 2024; Kondziella et al., 2020; Schankin & Goadsby, 2015; Solly, Clough, Foletta et al., 2021).

The potential comorbidity between VS and migraine raises the question: Do people with migraine *alone* have an increased susceptibility to the FP illusion? Unfortunately, these data do not answer this question conclusively. Although we found a main effect of Migraine, suggesting that individuals reporting migraine gave higher face scores to inanimate objects than individuals not reporting migraine, there was no evidence that control participants with migraine were more sensitive to the FP illusion than control participants without migraine (Figure 1D). That said, this discrete comparison may have been underpowered given the small number of participants in the control with migraine group (i.e., *N* = 22). The small sample size might also explain the results of the rank order analysis, where the data from the control with migraine group is the noisiest (see Figure 3A). Thus, future research targeting migraine groups is required to characterise sensitivity to the FP illusion in migraineurs. In this study, however, our specific aim was to investigate sensitivity to the FP illusion in VS syndrome. Results from the main analysis (Figure 1D) support the claim that people with VS are more susceptible to the FP illusion, even when the analysis was restricted to individuals without migraine (Figure 2A).

While face score has been used as a proxy for sensitivity to the FP illusion in a number of previous studies (Epihova et al., 2022; Robinson et al., 2025; Taubert et al., 2017; Taubert et al., 2022b; Taubert et al., 2023; Wardle et al., 2020; Wardle et al., 2022), further research is required to better contextualise the time course of the FP illusion in people with VS. For instance, the rapid prioritisation of real and illusory faces has been investigated in the past using detection paradigms (Caruana & Seymour, 2021; Jakobsen et al., 2023; Keys et al., 2021; Saurels et al., 2024). Importantly, detection paradigms in a laboratory setting would be less susceptible to the limitations associated with the present study, which include concerns about the accurate reporting of sensitivity to FP, the uneven recruitment of participants for each group, and the reliable collection of reaction time data. Even so, given the novelty of the current hypotheses tested herein, we argue that the risk the participants intentionally biased their responses is low.

As part of our pre-registered analysis plan, we investigated responses to individual images, averaging across participants. Using this approach, we were able to demonstrate that the scores given to the images by participants in the VS groups were systematically higher than the scores given to the same images by participants in the control groups (Figure 2B). A rank order analysis revealed the relative ease with which each object could be seen as a face was preserved across groups (Figure 3A). Thus, the people with VS syndrome did not respond erratically, nor is there strong evidence they accessed different visual cues than controls. Instead, the data indicate people with VS routinely assigned higher scores to examples of FP. Indeed, Figure 3A shows that, when scored by individuals in the VS groups, a large proportion of the objects received median face scores of 100 (i.e., the highest possible face score), implying that they were ‘extremely’ easy to see as faces. This was not the case for individuals in the control groups; only a small proportion of the objects received median face scores of 100 (Figure 3A). Interestingly, when we identified the images that elicited the largest differences in face score between the groups (Figure 3B), they were images that previous research has labelled as “difficult” to see as faces (Wardle et al., 2022). This suggests that ambient pictures of non-face objects that are difficult to see as faces could be employed as a diagnostic tool for VS syndrome that could complement self-report.

The FP illusion is a ubiquitous experience that, as primates, we are all familiar with. Nonetheless, differences in susceptibility to the FP illusion have been linked to a number of neurological (Akdeniz, Gumusyayla et al., 2020; Akdeniz, Vural et al., 2020; Uchiyama et al., 2012) and psychiatric (Abo Hamza et al., 2021; Caruana & Seymour, 2021; Romagnano et al., 2022) illnesses, in addition to pregnancy (Taubert et al., 2023). The discovery of a link between VS syndrome and the FP illusion supports the conjecture that VS is associated with the faulty resolution of visual ambiguity, either because (1) there is more noise intruding into the early stages of visual processing or (2) because the mechanisms for attenuating noise and distilling relevant cues in visual input are dysfunctional (Aeschlimann et al., 2024; Montoya et al., 2023; Silva & Puledda, 2023; Stern & Robertson, 2024). Thus, for people with VS, an initial error in face detection may be maintained or even enhanced during visual processing.
